# Supplementary material for: Ntrk1 Promotes Resistance to PD-1 Checkpoint Blockade in Mesenchymal Kras/p53 Mutant Lung Cancer
Source: Cancers (Basel). 2019 Apr 2;11(4):462. doi: 10.3390/cancers11040462 (PMC6521201; doi:10.3390/cancers11040462)
Supplement: Supplementary file 1 [file cancers-11-00462-s001.pdf]

Table S1. Positive and negative controls for FDAome dropout screen.

	393P						344P								
		veh			aPD1.t1			veh			aPD1.t1			aPD1.t2	
	tx.1	tx.2	tx.3	tx.1	tx.2	tx.3	tx.1	tx.2	tx.3	tx.1	tx.2	tx.3	tx.1	tx.2	tx.3
SSMD	1.74	1.80	1.68	2.17	1.86	1.84	2.15	1.32	1.45	1.21	1.85	1.14	1.07	2.14	1.07
LUC_CONTROL_199590	-0.71	1.14	1.52	0.56	2.95	1.76	3.77	2.49	1.35	0.24	0.31	-0.83	0.88	2.58	-0.23
LUC_CONTROL_199591	1.32	1.54	0.74	1.24	0.98	2.17	3.11	2.66	0.76	0.96	1.57	-2.12	-1.21	3.34	1.75
LUC_CONTROL_199592	6.1	2.07	2.04	2.51	2.49	0.93	1.07	2.24	2.39	0.92	1.59	0.44	2.15	0.33	0.35
LUC_CONTROL_199593	1.37	2	1.39	3.38	1.1	0.79	2.92	0.65	3.91	2.46	2.95	1.34	1.94	2.82	0.73
LUC_CONTROL_199594	3.59	0.84	0.85	3.11	4.76	0.23	0.56	2.09	2.33	2.92	1.37	1.86	8.58	1.72	2.41
LUC_CONTROL_199595	0.65	-0.08	0.43	2.61	1.11	3.5	0.76	2.23	1.11	1.41	1.1	2.8	1.94	1.18	1.46
LUC_CONTROL_199596	4.56	2.33	0.09	0.46	1.52	1.96	2.77	2.85	1.56	2.55	2.42	3.28	0.79	0.86	0.88
LUC_CONTROL_199597	3.9	2	0.56	1.86	3.63	0.85	3.19	2.37	1.66	3.37	0.76	3.59	1.07	1.97	1.26
LUC_CONTROL_199598	1.69	6.65	0.67	2.03	1.22	1.04	5.14	0.56	2.82	2.85	-0.14	-1.87	1.9	4.68	1.06
LUC_CONTROL_199599	2.73	-0.19	2.42	0.92	2.75	1.83	1.64	1.36	-0.18	0.5	3.41	-0.01	4.29	4.08	1.23
LUC_CONTROL_199600	0.04	0.31	1.41	-0.4	2.03	0.2	-0.2	-0.18	2.11	1.72	0.58	0.67	2.93	2.65	6.65
LUC_CONTROL_199601	1.56	0.79	2.27	2.35	0.15	0.62	1.79	0.87	1.75	0.41	0.25	0.48	0.98	2.92	0.85
LUC_CONTROL_199602	-0.63	-0.22	3.06	2.64	-1.07	0.06	-0.22	8.48	1.57	2.22	2.12	2.5	0.68	1.71	0.94
LUC_CONTROL_199603	-0.48	0.52	-0.19	-0.32	0.77	0.63	1.34	2.6	4.37	-0.98	0.94	-0.61	1.67	1.74	0.59
LUC_CONTROL_199604	-0.73	3.79	-0.43	2.02	2.81	0.35	1.74	0.19	4.01	0.27	0.04	1.39	1.62	0.06	0.67
LUC_CONTROL_199605	-0.58	0.28	-2.61	-2.05	-5.04	-1.39	0.43	-1.56	-0.67	-3.11	-2.35	4.16	0.21	2.1	0.24
LUC_CONTROL_199606	-0.9	-2.82	-2.38	-4.22	-3.46	-2.09	-4.72	-0.91	-1.98	-4.03	-0.48	-2.63	-6.1	-0.38	-5.54
LUC_CONTROL_199607	-0.73	2.21	-1.72	0.53	-5.62	-2.07	-3.97	-1.33	-4.85	-6.27	-2.49	-3.54	-1.52	0.4	-1.31
LUC_CONTROL_199608	-2.87	-0.66	-2.29	-2.57	-3.5	-5.97	-3.8	2.17	-2.95	1.45	-1.66	1.2	3.01	-2.92	-1.04
LUC_CONTROL_199609	-1.87	-1.82	-1.91	-2.81	-2.56	-2.95	-2.5	-2.08	-2.04	-0.97	-3.2	5.54	-0.82	-1.8	-1.93
Psma1_CONTROL_199570	0.51	-1.02	-0.84	1.58	2.88	-2.71	0.81	3.01	2.66	0.1	2.24	1.51	3.58	3.09	0.85
Psma1_CONTROL_199571	-0.66	-1.32	-0.37	-1.19	-1.69	-1.96	2.11	4.36	2.65	0.88	1.25	-0.09	2.61	1.17	0.21
Psma1_CONTROL_199572	0.98	-1.05	3.13	-0.54	-3.11	-0.15	2.36	5.37	2.46	1.12	0.61	4.93	-0.05	0.32	1.38
Psma1_CONTROL_199573	-0.83	1.53	1.56	0.03	-0.18	1.35	2.76	3.55	4.11	0.75	2.5	6.25	5.21	5.85	3.65
Psma1_CONTROL_199574	-0.89	0.47	-0.48	-2.59	-3.03	-1.12	1.57	2.95	0.85	0.76	-0.26	0.74	-0.04	2.83	-1.34
Psma1_CONTROL_199575	-2.64	-2	-0.95	-2.13	-3.87	-3.6	1.47	-1.66	-1.61	-1.15	-1.46	-3.05	-2.91	-3.27	-3.76
Psma1_CONTROL_199576	-3.55	-1.79	0.18	-2.74	-4.18	-2.35	-2.92	-2.7	-4.51	0.5	-2.18	-0.85	-5.81	-3.11	-1.86
Psma1_CONTROL_199577	-4.8	-2.82	-1.79	-1.57	-3	-0.14	0.13	-3.58	-3.03	-1.84	-2.25	-4.02	-0.29	-2.42	-0.81
Psma1_CONTROL_199578	-0.08	-5.12	-4.16	-3.26	-6.09	-3.09	-2.11	-3.93	-4.24	-6.77	-0.33	-6.2	0.39	-0.48	-5.61
Psma1_CONTROL_199579	-2.91	-0.44	-0.38	-1.69	-2.29	-2.65	-4.85	-3.26	-2.99	-5.3	-1.68	-6.53	-1.13	0	0.45
Psma1_CONTROL_199580	-4.72	-2.13	-2.64	-5.82	-0.94	-2.49	-3.57	-1.9	-4.62	-5.95	-7.09	-3.59	-3.57	-7.27	1.29
Psma1_CONTROL_199581	-3.52	-3.28	-5.27	-5.41	-1.84	0.29	-1.69	-2.96	-1.52	-1.81	-3.55	-2.26	1.9	-0.9	-2.17
Psma1_CONTROL_199582	-5.87	-1.23	-2.72	-5.26	-4.75	-6.22	-5.58	-4.22	-6.4	-4.45	-7.12	-7.83	-6.23	-4.68	-3.42
Psma1_CONTROL_199583	-5.34	-3.86	-0.52	-5.15	-6.23	-1.08	-3.46	-4.42	1.6	0.55	-4.66	-3.8	-0.95	-2.62	-0.99
Psma1_CONTROL_199584	-1.01	-3.19	-3.76	-0.22	0.08	-4.22	-1.38	-1.32	-2.65	1.72	-1.35	-2.88	-1.7	-1.29	-2.14
Psma1_CONTROL_199585	-3.29	-3.05	-3.47	-2.7	-3.94	-4.26	-2.73	-4.57	-0.97	-3.97	-0.57	-4.86	-2.85	-1.58	0.4
Psma1_CONTROL_199586	-2.18	0.11	-3.94	-2.53	-0.54	0.07	-4.38	-2.62	-2.28	-2.06	-4.06	-3.64	1.53	-0.41	-4.49
Psma1_CONTROL_199587	-0.24	-4.52	-1.25	-4.53	-5.49	-4.12	-3.43	-3.08	-1.18	-4.1	-4.37	1.43	-1.76	-3.32	-4.22
Psma1_CONTROL_199588	-1.8	-4.05	-0.99	-5.02	-4.04	-5.05	-4.19	3.73	-3.46	-5.54	-3.7	-4.33	-0.13	-4.69	3.82
Psma1_CONTROL_199589	-3.28	-2.51	-0.9	-3.52	-3.43	-3.35	-3.47	-1.19	-2.72	-2.88	-4.22	-1.93	-2.59	-2.01	-4.36
Rpl30_CONTROL_199550	-0.81	-4.14	-5.77	-6.16	-0.69	-6.78	-5.51	-5.46	-2.76	-4.44	-6.05	-6.02	-5.77	-5.83	-6.1
Rpl30_CONTROL_199551	-2.14	-5.94	-0.93	-2.31	0.02	-1.12	-3.42	-6.44	-4.89	-1.13	-3.35	-3.54	-4.03	-4.02	-1.04
Rpl30_CONTROL_199552	-4.19	-3.91	-4.4	-5.41	-1.09	-3.61	-2.19	-5.52	-2.58	-6.57	-2.12	-0.05	-4.77	-3.72	-0.46
Rpl30_CONTROL_199553	-2.83	-3.03	1.94	-3.96	-1.47	-1.83	-3.43	-4.08	-3.11	-7.37	-4.39	-2.57	-4.6	-5.47	-1.59
Rpl30_CONTROL_199554	-3.92	-7.03	-5	-4.75	-5.8	-6.9	-7.43	-2.59	-12.77	-8.7	-7.22	-6.41	-6.73	-6.16	-12.77
Rpl30_CONTROL_199555	-1.87	-4.96	-5.14	-2.77	1.16	-3.93	-4.17	-2.78	-4.67	-0.05	-2.2	-1.14	-1.75	-1.91	-4.01
Rpl30_CONTROL_199556	-7.67	-2.94	-3.88	-6.44	-6.87	-8.94	-4.57	-4.75	-6.46	-6.96	-4.06	-4.81	0.62	-6.16	-4.89
Rpl30_CONTROL_199557	-2.71	-1.29	-2.75	1.92	-2.47	-1.89	-5.33	-3.27	-0.23	0.09	-0.41	0.47	-2.05	-3.55	2.47
Rpl30_CONTROL_199558	-6.63	-5.08	-4.33	-4.34	-3.65	-4.26	-4.17	-4.39	-2.23	1.19	-4.21	-2.97	-3.59	0.24	-6
Rpl30_CONTROL_199559	-4.52	-2.33	-3.28	-3.02	-5.63	-4.35	-2.85	-2.68	-3.61	1.52	-2.13	1.47	-4.43	-3.21	-5.36
Rpl30_CONTROL_199560	-0.03	-0.01	-1.05	-1.94	-3.44	-2.3	-1.05	-1.82	4.11	-0.05	-0.58	6.85	6.05	1.26	-0.25
Rpl30_CONTROL_199561	-3.21	-2.31	-4.1	-2.88	-2.7	0.02	-2.3	0.56	-0.95	0.19	1.07	-0.83	1.04	-2.43	1.26
Rpl30_CONTROL_199562	-0.88	-1.98	-2.07	-3.34	-2.28	-4.45	-5.07	-4.86	-3.19	-3.83	-3.64	-2.35	2.45	0.04	-3.84
Rpl30_CONTROL_199563	-4.06	-0.73	-3.96	-1.55	2.06	-2.71	-0.07	-0.86	3.06	2.48	1.52	-1.25	-1.94	-5.65	-0.34
Rpl30_CONTROL_199564	0.14	-3.27	-4.46	0.43	-4.98	-4.21	-0.67	0.38	-1.94	2.74	-1.77	-0.33	1.46	-3.97	-3.07
Rpl30_CONTROL_199565	-2.85	-0.9	-1.94	-2.38	-3.75	-5.48	-2.86	-4.66	-2.74	-3.87	-5.52	-4.31	-1.58	-4.61	-3.86
Rpl30_CONTROL_199566	-5.6	-0.78	-11.54	-2	-5.76	-1.22	-6.57	-11.36	-11.36	0.95	-4.84	-4.01	-4.91	-3.18	-4.67
Rpl30_CONTROL_199567	-4.26	-2.49	-4.32	-3.43	-4.62	-0.79	-1.38	5.67	-1.69	-5.45	-4.04	-4.33	3.47	-2.59	3.93
Rpl30_CONTROL_199568	-3.87	-2.12	-4.44	-6.09	-0.7	-6.55	-3.16	3.75	-3.86	-5.84	-3.36	-6.31	-5.01	-4.93	-1.45
Rpl30_CONTROL_199569	-4.41	-1.18	-4.07	-0.78	-3.88	-0.82	-2.56	-0.6	1.51	-2.88	-2.69	-4.67	-2.26	-1.39	-5.29

Table S2. RSA values and rank scores for the FDAome shRNA dropout screen with PD-1 blocking antibody.

gene	393P veh logP	393P veh rank	393P aPD1.t1 logP	393P aPD1.t1 rank	344P veh logP	344P veh rank	344P aPD1.t1 logP	344P aPD1.t1 rank	344P aPD1.t2 logP	344P aPD1.t2 rank	model condition metric
Erbb2	-2.4	30	-3.6	14	-1.5	52	-2.0	34	-3.3	12	
Psmb1	-10.1	1	-8.1	2	-6.7	2	-6.4	1	-5.9	2	
Raf1	-9.3	3	-9.6	1	-2.0	24	-0.7	112	-0.3	161	
Pik3ca	-9.9	2	-5.3	6	-5.2	6	-3.6	10	-3.3	13	
Plk1	-2.7	23	-4.9	7	-3.3	13	-3.3	14	-3.3	10	
Wee1	-2.6	27	-2.7	21	-1.0	78	-0.7	108	-2.0	34	
Rac1	-6.2	5	-6.0	4	-5.7	4	-2.0	35	-1.5	48	
Mapk1	-3.4	15	-3.2	16	-1.1	74	-0.6	118	-0.4	145	
Cdk6	-2.6	26	-1.9	39	-5.3	5	-3.8	6	-2.8	22	
Mtor	-4.2	10	-4.0	11	-5.1	7	-3.6	9	-3.0	17	
Prkcd	-5.1	7	-3.9	12	-1.7	41	-2.1	30	-2.9	20	
Stat3	-4.9	8	-1.6	46	-4.9	8	-2.9	19	-3.2	14	
Bcl2	-4.1	11	-5.9	5	-4.2	9	-5.9	3	-6.3	1	
Pim1	-3.1	18	-1.3	55	-1.7	36	-2.6	21	-1.4	56	
Rrm1	-2.3	33	-2.7	22	-0.8	91	-0.9	92	-2.4	26	
Myc	-6.6	4	-3.7	13	-4.0	10	-1.8	37	-2.7	24	
Xpo1	-0.2	169	-2.0	36	-1.5	51	-2.4	25	-1.6	46	
Met	-3.1	17	-1.4	53	-1.1	73	-4.9	4	-1.0	86	
Esr1	-2.2	34	-3.1	17	-1.5	49	-2.3	26	-1.1	75	
Mapk8	-3.9	12	-1.6	44	-2.0	25	-3.7	7	-2.8	21	
Mapk7	-0.5	134	-0.6	117	-0.3	153	-0.8	106	-1.8	42	
Rarg	-4.3	9	-4.0	10	-3.3	14	-3.3	16	-1.0	94	
Jak2	-2.9	20	-2.4	28	-2.6	17	-4.1	5	-3.8	7	
Insr	-2.9	21	-4.5	9	-2.2	22	-1.2	68	-1.2	69	
Top2a	-0.6	108	-0.6	124	-1.1	72	-1.5	53	-0.8	107	
Psmd1	-5.8	6	-7.9	3	-6.7	3	-1.9	36	-3.5	8	
Ikbke	-3.7	13	-2.8	19	-0.7	101	-0.3	149	-0.8	106	
Egfr	-2.4	29	-2.1	31	-0.9	89	-2.6	22	-2.4	27	
Birc5	-2.0	37	-2.5	26	-1.8	33	-1.1	77	-2.3	30	
Blk	-0.7	95	-0.7	110	-0.1	187	-1.0	90	-0.4	138	
Pdgfrb	-1.0	69	-1.5	47	-1.0	80	-1.6	46	-1.5	49	
Src	-1.5	52	-1.5	51	-1.7	39	-0.2	176	-1.6	45	
Mcl1	-1.5	48	-0.7	101	-0.6	111	-1.7	44	-1.4	58	
Prkch	-0.4	150	-0.4	140	-0.4	150	-1.1	74	-0.8	105	
Top1	-2.4	32	-2.1	33	-1.7	37	-1.7	43	-3.0	18	
Cdk7	-0.9	78	-0.4	139	-3.7	12	-2.9	20	-2.2	31	
Cdk9	-2.0	39	-1.5	50	-0.7	107	-0.3	157	-0.2	169	
Nfkb1	-1.4	55	-0.5	134	-0.1	177	-0.4	144	-0.7	112	
Aurkb	-2.1	36	-2.0	35	-1.5	47	-1.6	47	-1.8	41	
Dot1l	-1.4	54	-2.2	29	-0.7	104	-1.1	73	-0.5	126	
Chek1	-1.8	41	-0.7	112	-1.4	55	-1.3	67	-3.9	6	
Prkdc	-1.1	62	-1.0	74	-1.6	42	-3.3	17	-1.2	68	
Flt4	-0.9	86	-0.7	103	-1.5	48	-2.2	27	-1.0	88	
Parp2	-0.4	136	-0.8	98	-0.4	148	-1.0	84	-0.5	125	
Pgd	-3.6	14	-1.7	42	-1.3	60	-1.1	76	-1.4	59	
Notch1	-1.7	43	-0.4	147	-0.5	135	-0.6	125	-1.2	74	

Atr	-2.2	35	-2.8	20	-1.3	62	-1.7	42	-2.1	33
Ehmt2	-1.8	40	-1.0	71	-0.2	163	-1.4	57	-1.1	80
Lap3	-0.6	110	-2.2	30	-0.4	140	-1.4	61	-0.4	140
Esr2	-2.7	24	-1.5	48	-0.6	128	-1.3	63	-1.9	35
Btk	-0.3	159	-0.5	128	-0.4	147	-0.5	128	-0.4	137
Fgfr4	-1.1	65	-1.0	79	-1.0	82	-1.4	60	-1.1	78
Gsk3a	-2.4	31	-1.0	73	-1.3	58	-0.5	129	-1.8	39
Kdm1a	-0.7	94	-0.7	104	-1.2	69	-1.3	64	-1.1	76
Drd2	-0.6	118	-0.5	136	-0.6	125	-0.1	186	-1.5	52
Trim24	-1.1	61	-1.1	66	-2.0	27	-1.0	83	-1.6	47
Braf	-0.3	158	-2.7	23	-0.9	85	-0.4	140	-0.5	131
Alk	-0.5	132	-0.9	82	-0.3	157	-0.1	183	-0.3	150
Prkcsh	-0.3	160	-0.1	175	-0.4	146	-0.2	174	-0.4	142
Epha2	-0.6	119	-0.9	86	-1.8	31	-1.3	65	-1.1	81
Whsc1	-0.7	98	-1.0	77	-1.3	57	-2.1	32	-1.9	37
Vegfa	-1.0	67	-1.1	69	-1.0	79	-0.7	115	-1.3	63
Pak4	-1.6	46	-0.8	99	-0.7	105	-2.1	28	-1.4	60
Pik3cg	-0.4	140	-1.2	59	-0.9	83	-0.8	105	-1.0	90
Ptpn11	-3.0	19	-2.9	18	-2.2	21	-2.5	23	-1.1	82
Mapk9	-0.7	100	-0.7	115	-0.6	130	-2.1	33	-1.7	44
Map4	-1.4	56	-1.2	58	-1.1	76	-3.5	11	-0.8	103
Cdk1	-0.9	83	-0.9	87	-1.6	43	-1.8	38	-1.3	62
Cdk2	-0.5	130	-0.8	95	-2.2	20	-2.5	24	-0.5	133
Hsp90aa1	-2.9	22	-1.1	67	-1.1	71	-3.7	8	-2.9	19
Mapk3	-0.4	148	-1.0	76	-0.6	123	-1.1	81	-0.2	167
Tnfsf11	-0.8	93	-0.6	125	-0.6	126	-1.0	89	-0.2	177
Eif4e	-2.7	25	-2.5	25	-3.0	15	-0.9	97	-1.3	64
Hdac1	-0.6	109	-1.3	54	-1.6	46	-1.8	40	-3.3	11
Prkcb	-0.4	141	-1.1	61	-0.1	183	-0.5	130	-0.2	182
Rxrb	-0.7	105	-1.1	62	-0.2	169	0.0	191	-0.2	178
Gls	-0.4	147	-0.7	114	-1.9	29	-0.4	147	-2.1	32
Ntrk1	-2.6	28	-0.9	84	-0.6	117	-3.4	13	-3.1	15
Prkca	-1.1	66	-0.6	118	-2.7	16	-1.7	45	-0.6	116
Txn1	-0.6	107	-1.3	56	-1.6	45	-1.5	52	-1.2	70
Nr2c2	-0.9	82	-0.8	91	-0.6	127	-0.6	119	-0.8	108
Parp1	-0.5	124	-1.0	78	-1.1	77	-1.2	69	-1.4	57
Ptgs2	-0.8	89	-0.7	113	-1.2	66	-0.5	127	-0.5	135
Fgfr1	-0.6	120	-0.4	137	-0.3	154	-1.2	72	-0.3	160
Rarb	-0.7	99	-1.0	72	-0.9	84	-0.9	98	-0.3	155
Gsk3b	-0.8	92	-0.9	90	-1.7	34	-0.3	164	-0.5	128
Tnf	-0.3	157	-0.4	145	-0.7	103	-1.7	41	-0.4	139
Rock2	-1.5	51	-2.1	34	-1.3	61	-1.6	50	-1.0	84
Ppm1d	-0.8	90	-0.8	92	-0.6	118	-1.5	56	-0.4	147
Irak4	-1.4	57	-0.2	168	-0.2	167	-1.3	62	-0.6	118
Ctnnb1	-1.5	50	-1.5	49	-1.7	38	-1.5	54	-2.7	23
Tubb4a	-0.5	127	-0.5	133	-1.7	35	-1.1	75	-3.0	16
Pik3cd	-1.6	45	-1.4	52	-0.6	120	-0.7	110	-1.2	67
Rps6kb1	0.0	184	-2.1	32	-0.1	178	-0.4	146	-1.0	85
Mapk11	-0.7	97	-0.3	153	-1.7	40	-1.0	87	-0.8	102

Mapk14	-0.9	84	-0.2	158	-0.7	109	-0.1	184	0.0	189
Brd4	-2.0	38	-2.6	24	-0.8	90	-1.0	82	-1.0	87
Aurkc	-0.5	131	-1.2	60	0.0	188	-0.4	135	-0.6	119
Map3k8	-0.3	163	-0.3	152	-0.2	164	-0.3	155	0.0	192
P4hb	-1.0	70	-0.4	143	-0.1	185	-0.4	136	-1.4	61
Ldha	-0.5	129	-1.1	65	-2.1	23	-1.2	70	-1.2	71
Aurka	-1.3	58	-0.9	88	-0.6	110	-1.4	58	-2.3	29
Parp3	-0.9	85	-1.7	43	-2.0	26	-1.3	66	-1.9	36
Il6ra	-0.5	125	-0.9	85	-0.8	96	-0.9	96	-1.0	89
Fgr	-0.6	113	-1.0	80	-1.3	59	-0.6	123	-0.9	96
Ms4a1	-0.6	114	-0.3	148	-0.1	179	-0.1	188	-0.2	174
Fyn	-0.7	96	-0.4	142	-0.3	159	-0.4	138	-0.6	123
Hdac3	-0.5	123	-0.1	179	-0.8	97	-0.2	179	-0.1	188
Kit	-1.0	73	-0.2	157	-1.3	63	-1.0	88	-0.3	156
Akt1	-0.1	175	-0.7	102	-0.8	98	-0.1	187	-0.2	171
Ptk2	-1.8	42	-2.0	37	-3.9	11	-3.3	15	-4.6	4
Tyro3	-0.3	155	-0.3	154	-1.8	32	-0.3	163	-1.5	51
Jak1	-0.6	117	-0.5	130	-1.9	28	-0.7	111	-0.6	120
Idh1	-0.3	156	-0.2	166	-0.6	113	-0.2	173	-0.4	141
Lyn	-1.0	71	-3.3	15	-0.6	121	-0.9	100	-2.3	28
Cdk4	-3.2	16	-4.7	8	-7.9	1	-6.1	2	-4.6	3
Kdr	-0.1	176	-0.1	173	-0.1	181	-0.3	161	-0.2	170
Ptpn6	-1.0	75	-2.4	27	-1.6	44	-0.3	153	-4.4	5
Ezh2	-0.5	121	-1.1	63	-1.5	50	-0.8	104	-0.6	117
Xiap	-0.5	133	-1.1	68	-0.3	158	-0.7	107	-0.2	163
Ptch1	-0.4	137	-0.4	146	-1.2	70	-1.8	39	-0.8	99
Tec	-1.6	47	-0.8	93	-0.6	114	-3.4	12	-1.7	43
Fgfr2	-0.9	77	-2.0	38	-0.9	87	-1.4	59	-1.8	40
Chek2	-0.4	145	-0.4	141	-1.4	53	-1.6	48	-0.9	95
Mapk12	-0.7	101	-0.7	108	-1.4	56	-0.3	160	-0.6	121
Rxra	-0.6	111	-1.0	70	-2.4	18	-0.5	131	-2.5	25
Frk	-0.3	154	-0.1	181	-0.2	162	-0.2	178	-1.3	66
Hdac6	-0.6	106	-0.9	83	-1.2	68	-0.6	121	-1.2	73
Akt3	-0.9	81	-0.8	94	-0.8	93	-0.6	126	-0.7	113
Mknk1	-0.3	162	-0.7	105	-0.4	144	-0.1	182	-0.5	129
Top2b	-1.1	64	-0.5	126	-1.4	54	-0.3	156	-1.4	53
Casp3	-1.0	68	-0.9	81	-0.7	102	-0.2	168	-0.8	100
Flt1	-0.8	91	-0.5	132	-1.0	81	-1.1	79	-1.2	72
Prkaa1	-0.6	115	-0.7	107	-0.5	137	-2.1	31	-0.1	187
Hdac8	-1.0	76	-0.7	109	-0.2	166	-0.8	103	-1.4	55
Map2	-0.3	161	-0.3	149	-0.4	151	-0.2	177	-0.2	162
Tek	-0.1	173	-0.1	169	-0.2	170	-0.9	99	-0.3	153
Hdac2	-1.0	74	-1.7	41	-0.5	133	-0.2	170	-0.4	143
Ccr5	-0.9	80	-0.6	121	-0.8	100	-0.3	148	-0.4	148
Tnfrsf8	0.0	180	-0.1	183	-0.5	139	-0.4	143	-0.3	158
Axl	-0.5	126	-0.1	170	-1.2	67	-0.9	94	-0.8	101
Atm	-0.2	172	-1.1	64	-0.4	149	-0.4	145	-0.2	176
Cd274	-0.6	116	-0.7	100	-0.3	155	-0.2	167	-1.1	77
Prkcq	-0.5	122	-0.5	131	-0.1	182	-0.2	171	-0.3	157

Dhfr	0.0	190	-0.6	119	-0.6	119	-0.2	175	-0.5	127
Pim3	-0.2	167	-0.6	122	-0.4	152	-0.2	166	-1.0	92
Il1b	-1.1	63	-1.7	40	-0.9	86	-2.1	29	-1.0	93
Mapk13	-0.3	152	-0.6	116	-0.7	106	-1.0	86	-1.1	83
Tyms	0.0	187	-0.4	144	-0.2	165	-0.7	109	-0.1	185
Map2k2	-0.7	104	-0.2	164	-0.6	122	-0.4	137	-0.7	114
Prkci	-1.7	44	-1.6	45	-0.6	124	-0.5	134	-1.1	79
Map3k14	-0.9	79	-0.2	163	-1.2	65	-0.6	122	-0.5	132
Flt3	-0.7	103	-0.6	123	-1.8	30	-0.3	150	-3.4	9
Prkcg	-1.2	59	-0.9	89	-0.8	95	-0.9	93	-0.9	98
Pim2	0.0	189	-0.1	185	0.0	192	0.0	190	0.0	190
Smo	-1.0	72	-0.8	97	-0.6	115	-0.4	142	-1.4	54
Igf1r	-0.1	178	-0.1	180	-0.2	168	-0.1	189	-0.2	164
Sgk3	-0.4	144	-0.2	161	-0.1	180	-0.5	133	-0.2	172
Rara	-0.2	164	-0.3	150	-0.6	112	-0.7	116	-0.7	115
Il6	-0.5	128	-0.8	96	-1.2	64	-1.6	49	-0.2	175
Jak3	-0.2	170	-0.2	160	-0.6	129	-0.6	124	-0.7	111
Bcr	-0.4	146	-0.7	111	-0.2	173	-0.1	180	-0.2	181
Pdgfra	-1.2	60	-0.5	127	-0.5	131	-0.9	95	-0.8	104
Tbk1	-0.4	142	-0.6	120	-0.2	171	-0.5	132	-0.4	149
Fgfr3	-0.8	88	-0.5	135	-0.4	141	-0.8	102	-1.8	38
Tnfsf13b	-0.7	102	-0.1	177	-0.1	174	-1.1	80	-0.1	184
Prkcz	-0.2	171	-0.2	165	-1.1	75	-0.8	101	-0.4	144
Ar	-0.8	87	-0.1	186	-0.6	116	-0.3	159	-0.3	159
Prkce	-0.4	151	-1.3	57	-0.9	88	-0.2	172	-0.3	152
Abl1	0.0	182	-0.5	129	-0.2	172	-0.6	117	-0.5	134
Abl2	0.0	191	0.0	190	-0.8	99	-0.4	139	-0.4	146
Trpv1	0.0	181	-0.1	188	-0.7	108	-0.2	169	-0.5	130
Nampt	-0.4	149	-0.2	167	-0.5	138	-1.5	55	-0.6	124
Pdk1	0.0	185	-0.2	159	-0.8	92	-1.2	71	-0.7	110
Pak1	-0.6	112	-0.4	138	-0.4	142	-0.3	162	-1.5	50
Mapt	-0.2	165	-0.1	176	-0.3	160	-1.6	51	-1.0	91
Porcn	-1.5	49	-0.2	156	-0.5	132	-0.3	158	-0.2	183
Rock1	-0.2	166	-0.1	174	-0.1	175	-3.1	18	-0.3	154
Itk	-0.4	138	-0.1	171	-2.3	19	-1.1	78	-0.5	136
Map2k1	-0.1	179	-0.1	182	-0.3	156	-0.3	165	-0.3	151
Ret	-0.3	153	-0.1	184	0.0	190	-0.1	185	-0.2	166
Idh2	-0.4	139	0.0	191	0.0	191	-0.3	151	-0.2	179
Cd52	-0.1	174	-0.3	155	0.0	189	0.0	192	-0.2	173
Pik3cb	0.0	192	-0.3	151	-0.4	145	-0.4	141	-0.8	109
Akt2	-1.4	53	-0.7	106	-0.5	134	-0.3	152	-1.3	65
Nudt1	-0.2	168	0.0	189	-0.3	161	-0.7	113	-0.2	165
Syk	0.0	183	-1.0	75	-0.1	176	-0.9	91	0.0	191
Pigf	-0.1	177	-0.1	178	-0.1	186	-0.7	114	-0.2	168
Mdm2	0.0	186	-0.1	187	-0.1	184	-1.0	85	-0.2	180
Lck	0.0	188	0.0	192	-0.4	143	-0.3	154	-0.1	186
Bmx	-0.4	143	-0.1	172	-0.5	136	-0.1	181	-0.9	97
Crebbp	-0.5	135	-0.2	162	-0.8	94	-0.6	120	-0.6	122

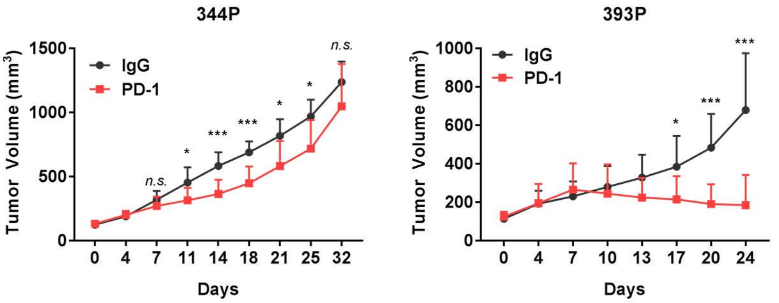

Figure S1. 344P and 393P responses to anti-PD-1 treatment *in vivo*. 344P mesenchymal cells (left) or 393P epithelial cells (right) were implanted subcutaneously into 129/Sv wildtype mice. Once tumors reached ~200mm^3, they were treated with either IgG control or PD-1 blocking antibody. Tumor growth was measured using calipers every 3-4 days after initiation of treatment.

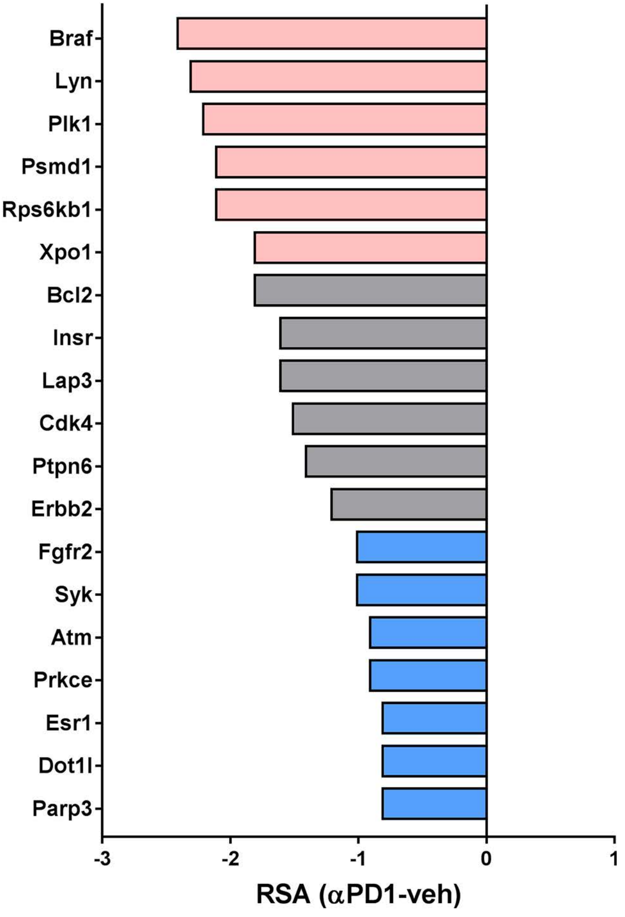

Figure S2. Ntrk1 shRNA does not demonstrate significant dropout from 393P tumors treated with anti-PD-1 antibody. Results from FDAome shRNA dropout screen in the 393P tumors graphed as a differential score. The RSA from the isotype treatment condition for each gene was subtracted from the RSA from the same gene in the anti-PD-1 treatment group as described in Figure 2A.

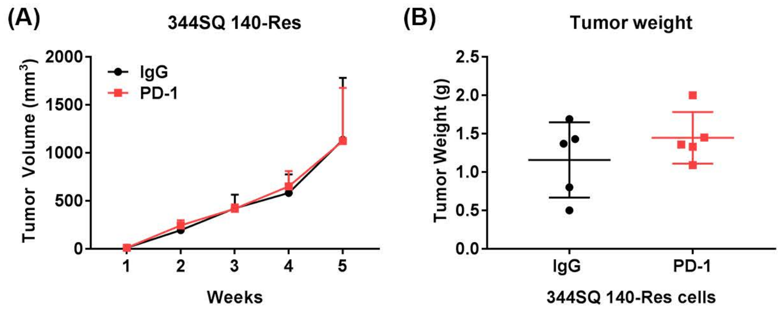

Figure S3. Cell lines derived from anti-PD-1 resistant tumors maintain resistance when re-challenged with anti-PD-1 antibody in vivo. (**A**) 344SQ tumors treated with PD-1 blocking antibody were excised after PD-1 resistance (~week 6-7). Tumors were then processed for primary cells to generate anti-PD-1 resistant lines. One of these lines, the 344SQ 140-Res cells, were then re-implanted subcutaneously into wildtype mice and treated with either isotype control or anti-PD-1 antibody. Tumor growth was measured weekly by calipers. (**B**) After 5 weeks, the mice from panel A were euthanized, and tumor weights were recorded at the time of euthanasia. n = 5 mice.

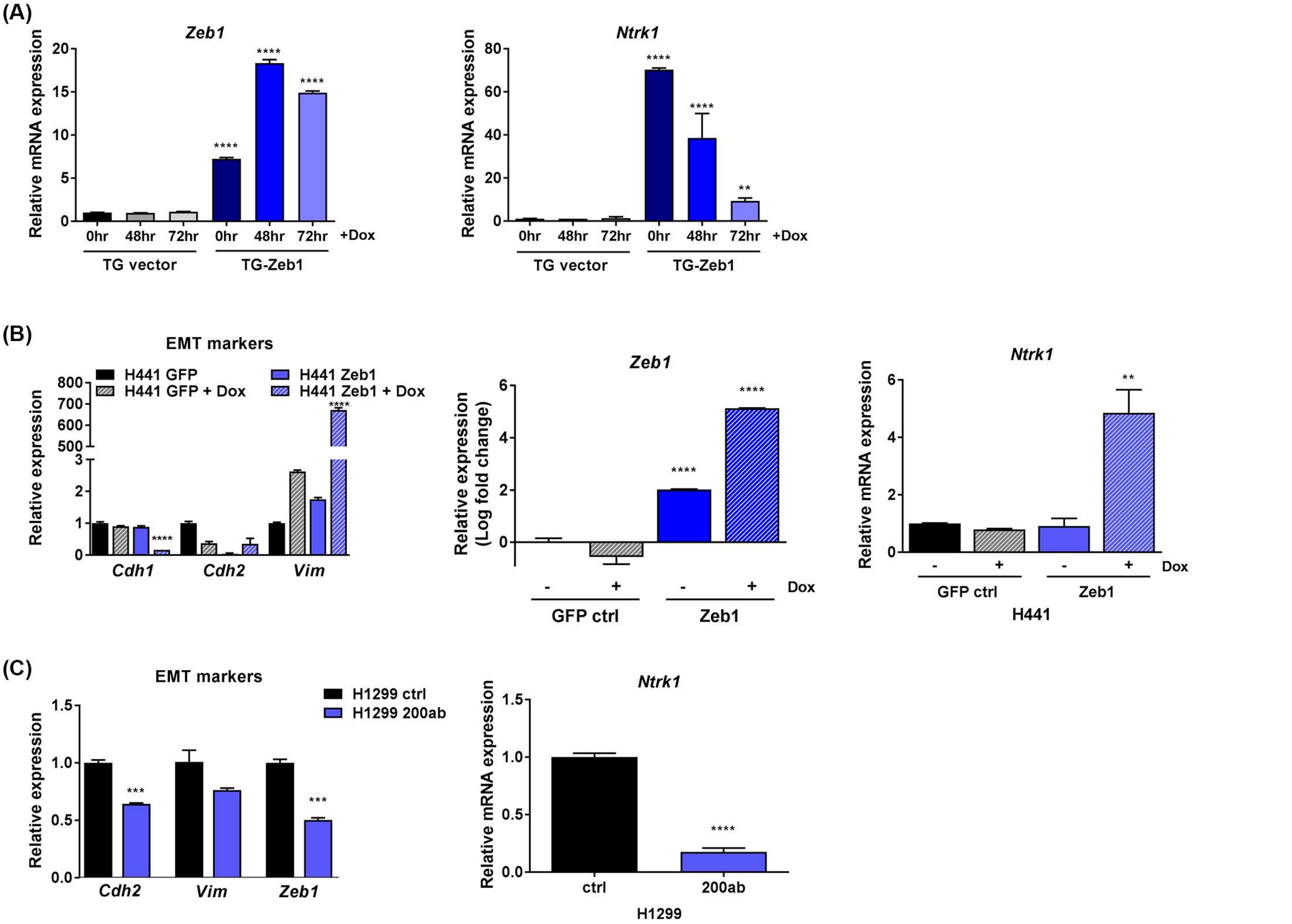

Figure S4. Ntrk1 expression correlates with the mesenchymal status of cells. (**A**) 393P cells stably expressing a doxycycline-inducible GFP-Zeb1 or GFP control were cultured for 48 and 72hrs in media containing 2μg/ml doxycycline. RNA was extracted from these cells to analyze both Zeb1 expression (left) and Ntrk1 expression (right) using real-time qPCR. (**B**) Human H441 lung cancer cells expressing Zeb1 under a doxycycline-inducible promoter were analyzed using qPCR for expression of mesenchymal markers (Cdh2, Vim, and Zeb1) and the epithelial marker Cdh1. Ntrk1 expression (right graph) was also analyzed using qPCR in these cells. (**C**) Human H1299 cells expressing an inducible miR-200ab vector were assayed for EMT marker expression via qPCR with addition of doxycycline as described in panel B. The expression of Ntrk1 in these cells was also analyzed (right).

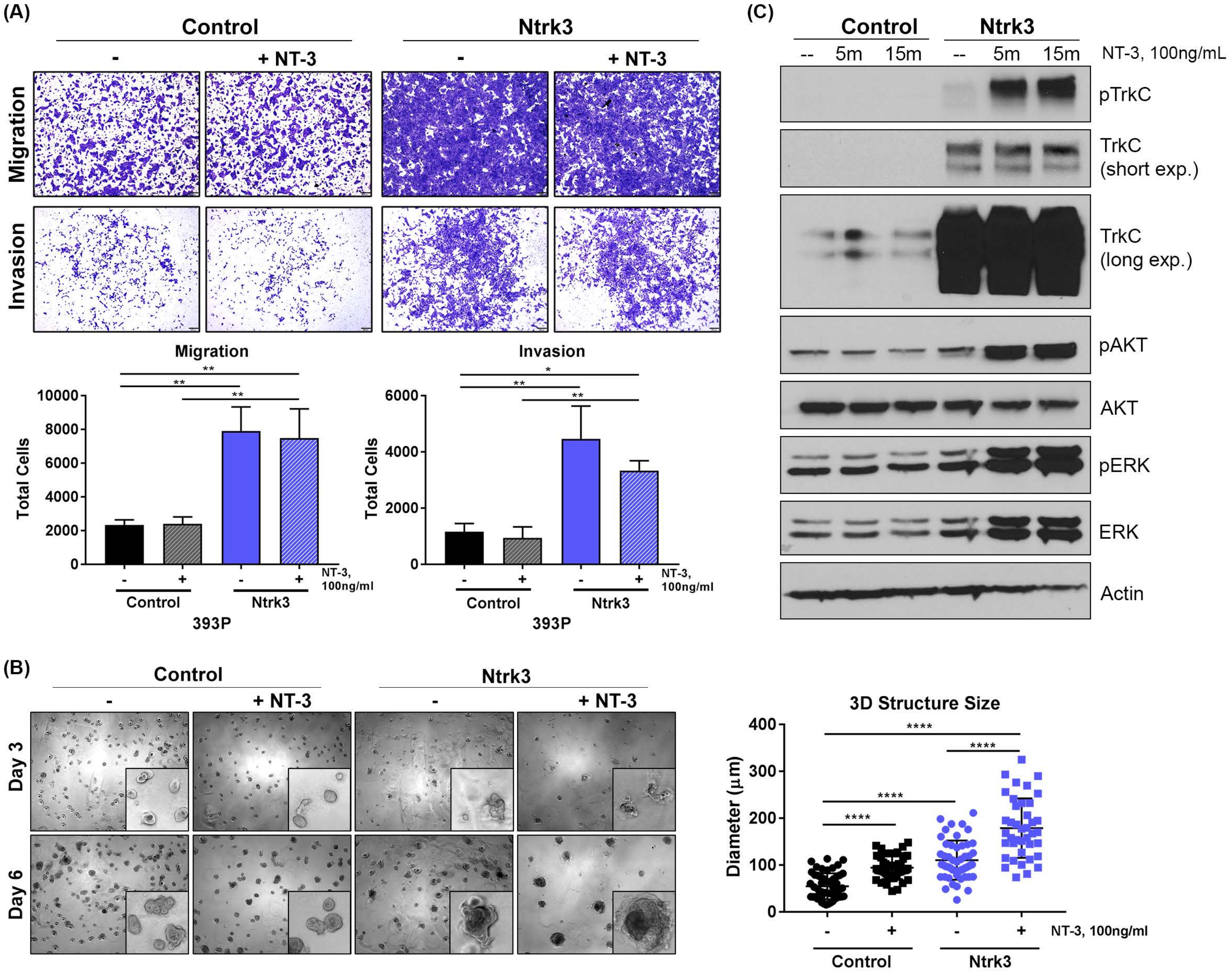

Figure S5. Overexpression of Ntrk3 increases cell migration, invasion, 3D growth and signaling via MAPK and AKT pathways. (**A**) 393P control or Ntrk3 overexpressing cells were plated in transwell inserts to measure migration (transwell inserts without Matrigel) or invasion (inserts with Matrigel). Exogenous NT-3 was added as indicated at a concentration of 100ng/ml. Representative images of inserts stained with crystal violet are shown for each condition, and an average of 3 inserts per condition is graphed below. (**B**) 393P Ntrk3 overexpression cells were plated as single cells on a layer of Matrigel and grown for 6 days. NT-3 was added at the time of seeding. Representative images of structures are shown at days 3 and 6. Day 6 structure diameter was quantified using ImageJ and graphed to the right. n=40-50 cells per condition. (**C**) 393P Ntrk3 overexpression or control cells were stimulated with NT-3 ligand for 5 and 15 minutes. Cells were then lysed and assayed for phospho-TrkC expression and downstream signaling via Western blot.

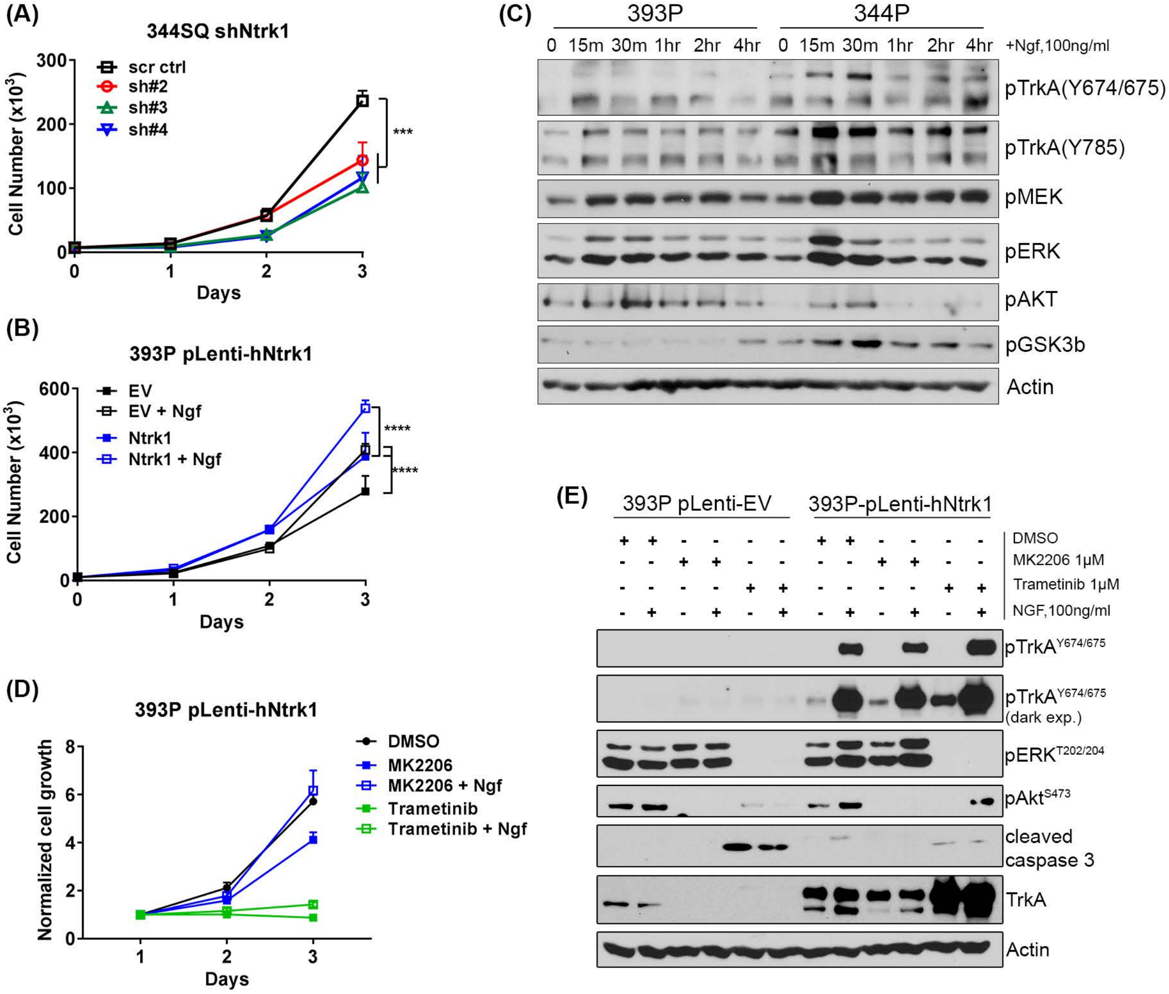

Figure S6. Ntrk1 promotes cellular growth through MAPK signaling. (**A**) 344SQ cells with Ntrk1 knockdown or were plated at equal cell numbers on day 0, then counting every 24 hours for 3 days and compared to scrambled control cells. Trypan blue exclusion was used to determine viable cells. (**B**) Cell growth was assayed in 393P Ntrk1 overexpression cells compared to empty vector control cells as described in panel A. NGF was added at a concentration of 100ng/ml at the time of seeding. (**C**) 393P and 344P cells were stimulated with NGF ligand at 100ng/ml for a short time course. Cells were lysed at the indicated time point and protein analyzed via Western blot for phospho-TrkA activation as well as downstream signaling cascades. (**D**) 393P empty vector control or Ntrk1 overexpression cells were plated with either DMSO control, MK2206 AKT inhibitor at 1uM, or trametinib at 1uM. Additionally, NGF was added to the culture where indicated. Growth was measured using MTT assay and normalized to DMSO absorbance average on Day 1. (**E**) 393P Ntrk1 or control cells were plated with AKT or MEK inhibitors as described in panel D. After 48 hours of treatment, Western blot analysis was performed for phospho-TrkA expression. Phospho-ERK was used to demonstrate efficacy of trametinib whereas phospho-AKT shows efficacy of MK2206. Cleaved caspase 3 was used as a marker of cellular death via apoptosis.

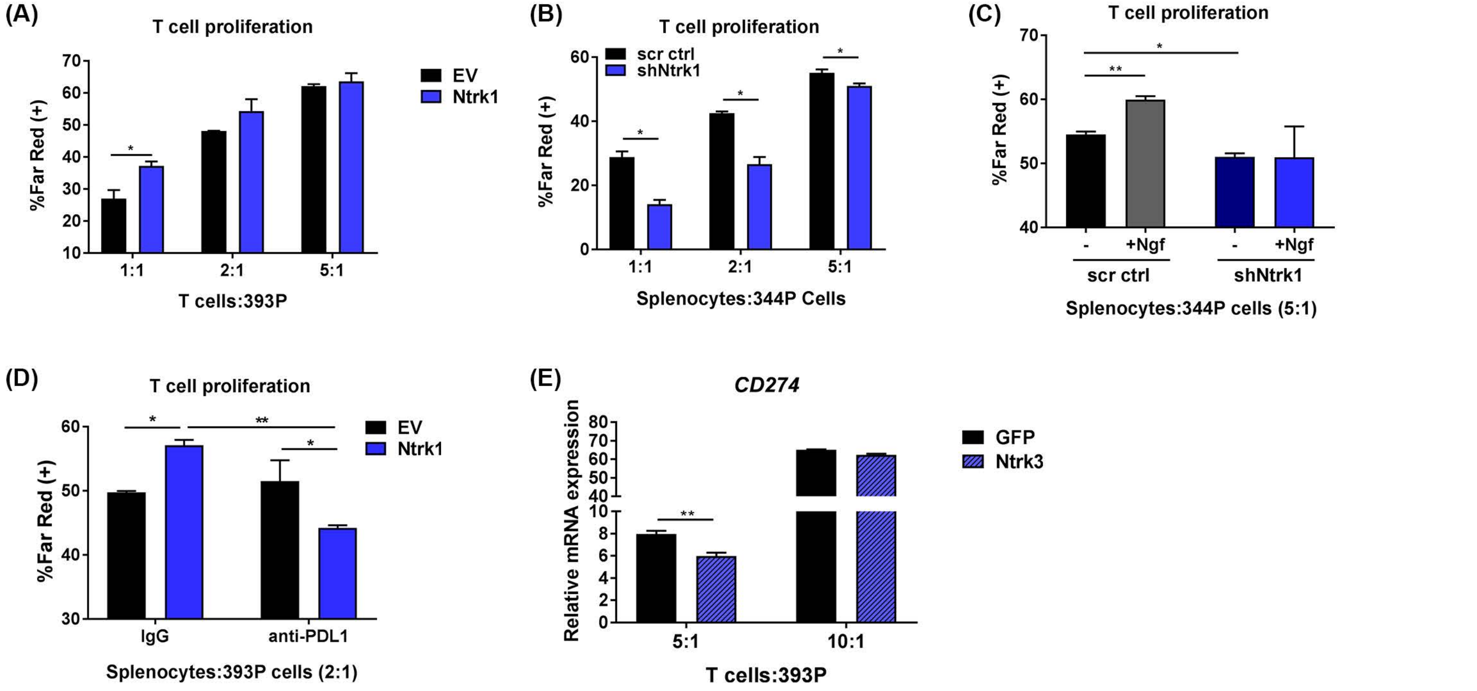

Figure S7. T cell proliferative capacity is inhibited as a function of Ntrk1 expression through PD-L1 activity. (**A**) Total splenocyte cells were labelled with a far red proliferative dye and plated with 393P cells overexpressing Ntrk1 at increasing ratios. After 4 days, T cells were collected and analyzed for far red expression using flow cytometry, with less positive signal of the dye indicating a greater degree of proliferation. (**B**) T cell proliferation when co-cultured with Ntrk1 knockdown cells was analyzed as described in panel A. (**C**) 344P cells expressing either scrambled control or Ntrk1 specific shRNA were co-cultured with total splenocytes as described in panel B. Exogenous NGF was added to the co-culture at a concentration of 100ng/ml. (**D**) 393P Ntrk1 overexpression or vector control cells were co-cultured with total splenocytes. At the time of seeding, anti-PD-L1 antibody or an IgG control antibody was added to the co-culture at a concentration of 20ug/ml. T cell proliferation was measured using flow cytometry analysis for far red proliferation staining. (**E**) 393P Ntrk3 overexpression cells or GFP control cells were co-cultured with total splenocytes at 5:1 and 10:1 ratios as described in panel A. After 4 days, splenocytes were washed out and RNA extracted from the tumor cells. Real time qPCR analysis was then used to examine the expression of CD274 in the tumor cells.

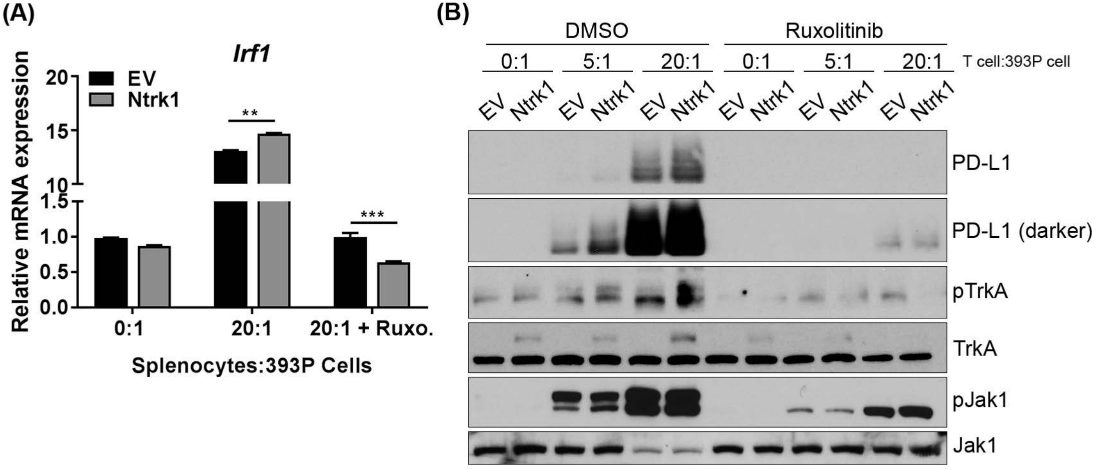

Figure S8. Treatment with a Jak1/2 inhibitor blocks upregulation of Irf1 and PD-L1 in splenocyte co-culture. (**A**) 393P Ntrk1 and EV cells were plated alone or in co-culture with splenocytes at a 20:1 ratio. Ruxolitinib was added to samples to inhibit Jak1/2. After 3 days, samples for collected for RNA and qPCR was completed to analyze the expression of Irf1. (**B**) A co-culture assay with 393P Ntrk1 and EV cells was completed as described in A, with 5:1 and 20:1 ratios. After 3 days of co-culture and treatment with Ruxolitinib, samples were collected for western blot analysis of phospho-TrkA, phospho-Jak1, and PD-L1 expression.
